# Supplementary material for: Survival of musical activities. When do young people stop making music?
Source: PLoS One. 2021 Nov 24;16(11):e0259105. doi: 10.1371/journal.pone.0259105 (PMC8612519; doi:10.1371/journal.pone.0259105)
Supplement: S1 Table — (DOCX) [file pone.0259105.s001.docx]

**S1 Table. Self-reported most played instruments of first-time participants.**

| Instrument | *n* |
| --- | --- |
| Vocals | 771 |
| Piano | 482 |
| Guitar | 590 |
| Drums | 249 |
| Xylophone | 125 |
| Flute | 26 |
| Oboe | 232 |
| Clarinet | 11 |
| Bassoon | 47 |
| Trumpet | 2 |
| Trombone | 29 |
| Tuba | 21 |
| Saxophone | 2 |
| Horn | 54 |
| Violin | 9 |
| Cello | 105 |
| Viola | 60 |
| Double bass | 11 |
| Harp | 12 |
| Others | 15 |
| No instrument | 179 |
