# Supplementary material for: Survival of musical activities. When do young people stop making music?
Source: PLoS One. 2021 Nov 24;16(11):e0259105. doi: 10.1371/journal.pone.0259105 (PMC8612519; doi:10.1371/journal.pone.0259105)
Supplement: S2 Table — N = 3,915, all items were measured on 7-point Likert scales, ranging from 1 (Disagree strongly) to 7 (Agree strongly), adjectives in italic were added to the original scale. (DOCX) [file pone.0259105.s002.docx]

## S2 Table. Ten Item Personality Inventory (TIPI; Gosling, et al., 2003) – extended.

| Personality trait | Item (I see myself as…) | M | SD |
| --- | --- | --- | --- |
| Openness | open to new experiences, complex + *curious, thoughtful* | 5.16 | 1.16 |
|  | conventional, uncreative + *shallow, simple* |  |  |
| Conscientiousness | dependable, self-disciplined + *responsible, persistent* | 4.93 | 1.22 |
|  | disorganized, careless + *lazy, irresponsible* |  |  |
| Extraversion | extraverted, enthusiastic + *sociable, lively* | 4.72 | 1.22 |
|  | reserved, quiet + *shy, private* |  |  |
| Agreeableness | sympathetic, warm + *kind, patient* | 4.79 | 1.36 |
|  | critical, quarrelsome + *grumpy, selfish* |  |  |
| Emotional Stability | anxious, easily upset + *touchy, fearful* | 5.16 | 1.31 |
|  | calm, emotionally stable + *independent, peaceful* |  |  |

*N* = 3,915, all items were measured on 7-point Likert scales, ranging from 1 (Disagree strongly) to 7 (Agree strongly), adjectives in italic were added to the original scale.
