# Supplementary material for: Survival of musical activities. When do young people stop making music?
Source: PLoS One. 2021 Nov 24;16(11):e0259105. doi: 10.1371/journal.pone.0259105 (PMC8612519; doi:10.1371/journal.pone.0259105)
Supplement: Attachment — Submitted filename: Rebutal_musical survivial.docx [file pone.0259105.s003.docx]

Reviewer #1: With shrinking space for art education at school in most Western countries it is becoming increasingly important to examine factors that stimulate sustenance of art habits for life. The present contribution is important from this point of view.

- Thank you. We very much agree.

In the art research field it is very difficult to find all the relevant literature studies published in all kinds of scientific journals. I have with coworkers done one of the few studies examining factors that make children continue music practicing as adults but the authors have obviously not seen it, maybe because it was published in a paediatric journal in which one would not expect to find such an article. However, it examines many of the questions that the authors are addressing so it is relevant (Acta Paediatrica 104 :274-284, 2015). It was a quasi-prospective study based upon a large Swedish twin cohort (with random exclusion of one member of each twin pair – to equate it with a normal population study) with 3820 participants aged 27-54 who were asked to reconstruct number of training hours as well as a number of other relevant conditions during different age periods. Some of the conclusions are similar to the ones that the authors are making here, despite the fact that widely different statistical analyses were performed. 70% had had some exposure to music teaching added to the regular school curriculum, and almost exactly as the authors report in the present study only half of those subjects continued active music practice as adults. The likelihood of sustained music practice up to adult years decreased with start of extra music. The calculation in the Swedish study was that for every year of delayed start of extra music between age 6 and 17 there was a 7% decrease in likelihood that the participant would continue music practice up to adult years. It would be interesting if the authors could do a similar calculation in the present study. It is an important perspective because the Swedish findings mean that early start is very important.

- Thanks for pointing us to the very interesting and important study. We incorporated the reference now. Indeed, these are a very fitting findings and a very useful analysis. We included the musical starting age as a predictor now in our study, too. However, we did not find it to be a significant predictor. We discuss this in more detail but would argue that the musical home environment variable is a closely related factor which is highly correlated with starting age and therefore lessens the impact of starting age. We compared our results to those of your study in the discussion now.

As in the present study, there were fewer boys who started extra music, but on the other hand those boys who did were more likely than girls to keep their habit. This is similar to the present study. As in the present study genre was important as well as instrument – but the findings for piano and guitar were widely diverging in the two studies. Music and other cultural activities in the families were important survival factors as were special public music schools (which were attended by few and probably talented) but also community cultural schools for leisure art learning (much larger numbers) increased survival slightly.
In the Swedish study in which sampling represented a much longer period it was also possible to examine a cohort effect – participants continued active music practice as adults to a much smaller extent if they went to school during the 2000 :s than if they did so during the late 1970 :s.
I mention this because it would be really nice if these two studies although they have different research questions and are quite different in design could be compared in the discussion. It would also be interesting if the authors could refer to the Swedish twin studies (Ullén has been PI) which have explored the role of genetic factors which the authors also discuss. For instance pleiotropy may play a role in several of these relationships.

- Thanks for pointing this out. We included the Swedish twin study and the genetic factors in our discussion.

The authors mention in the discussion that they feel that the cross-sectional analysis has advantages. But it also has disadvantages : We do not know whether a high TOM score is secondary to a failure in music practice or whether it is a true predictor (rationalization retrospectively : I now understand that I failed because musical talent cannot be taught). Their discussion around conscientiousness is interesting, but the cross-sectional design makes it impossible to know whether it is causal. The family culture factor however seems very established and is important also in the Swedish study.

However, in general this is an important study.

- Thank you very much. The incorporation of the studies you have mentioned greatly improved the manuscript and we hope it finds your approval.

Reviewer #2: This paper included a survival analysis to document ages at which student participation in music declines. The authors utilized a longitudinal study of students 10-17 years of age and found that home musical environment was an important factor in whether students persisted in the latter ages. While the authors state other papers have provided a rationale to "why" students may choose not to persist in continuing music performance and stated that may be in forthcoming studies, I found myself wanting to link the ages to the rationale for the choice to forego continuing in music.

- Thanks for your summary and evaluation. We agree that age is an important factor and we discussed this in more detail now. Following reviewer 1’s comments, too we also incorporated starting age as a predictor now.

Specific concerns in the paper include the use of "(e.g., [citation])" when the "e.g." felt unwarranted
There are several typographical errors in the paper that can be remedied quickly
P 9, L 197: Write out the citation for #34
P10, L 223: revise "Table6Table 5"
P 12, L 259: remove redundant "scale"
P 15, 325: revise "that boys have a are less likely"

- We corrected the mistakes and deleted the “.e.g.”s

Discussion: there are other likely rationale besides puberty for decisions not to persist in music. In the US (understood this study did not originate there), athletics are in higher demand for time as well as employment, and often conflict with musical requirements. Many studies find those conflicts are a leading cause is attrition in music programs. Is there a way to connect your work with those findings? I think that combination would truly show the decline is more than anecdotal, as your data show.

- This is a very interesting point. Indeed, the study did not originate in the US. Linking musical dropouts to other activities like athletics would be very interesting. Unfortunately, we only have little data on physical education and nothing on athletic programs or activities. Therefore, we cannot accomplish the desired analyses. However, we included this promising rationale in our discussion as an additional explanation and as a suggestion for future studies.

None of the Figures have captions which makes it hard for the reader to connect the writing to the information (aside from very small print). This is the most significant need to address.

- Thanks for pointing this out. We think the Figures are added automatically to the document as we uploaded them separately. We inserted captions in the manuscript. Hopefully, they will be added in the final version.
