# Supplementary material for: Survival of musical activities. When do young people stop making music?
Source: PLoS One. 2021 Nov 24;16(11):e0259105. doi: 10.1371/journal.pone.0259105 (PMC8612519; doi:10.1371/journal.pone.0259105)
Supplement: Attachment — Submitted filename: Rebutal_musical survivial.docx [file pone.0259105.s004.docx]

4. Have the authors made all data underlying the findings in their manuscript fully available?

The PLOS Data policy requires authors to make all data underlying the findings described in their manuscript fully available without restriction, with rare exception (please refer to the Data Availability Statement in the manuscript PDF file). The data should be provided as part of the manuscript or its supporting information, or deposited to a public repository. For example, in addition to summary statistics, the data points behind means, medians and variance measures should be available. If there are restrictions on publicly sharing data—e.g. participant privacy or use of data from a third party—those must be specified.

Reviewer #1: No

Reviewer #2: No

- Since we collected data from students, we can only share data upon request. Researchers will have to agree to a data protection agreement. We included a statement on this now.

6. Review Comments to the Author

Reviewer #1: The authors have adequately dealt with the comments

I cannot find any information regarding accessibility of data for other researchers but I may not have looked for it sufficiently. However, there is reason to believe that the authors will give access to their data

- We included a section on this now (see above)

Reviewer #2: The revision is much improved and I thank the authors for their time in addressing the concerns. My remaining issues are minor and can be addressed quickly, I believe. They are summarized below:

P 11, L234: "Table6Table5" is still included in the sentence and should be edited to reflect the correct reference

- Thanks, we corrected this now.

P20, L432: you state "..several studies [4]" and cite only the one. Please reflect multiple studies.

- You are right. We revised this.

P21, L453: suggest moving the r and p values after "with starting age" to make it easier for the reader.

- Done.

P21, second paragraph: the authors argue that conscientious pupils focus on school and drop leisure activities, but it could also be argued that a high level of conscientiousness may lead to dropping music due to not feeling successful in performing on the instrument of choice

- Thanks for pointing this out. We included this as an additional explanation now.

P22, 480: if only a small proportion of students participated more than once, can you truly call it a "longitudinal study". Rather, it is more of a sample across ages. Perhaps remove "longitudinal" since that could be misleading

- You are right. Since this is part of a longitudinal project which will run for several more years, we always talk about a longitudinal study. But we clarified this in the discussion now.

P23, LL516-518: evaluating genetic factors to explain relationships is a large leap to take in your study, given that single added citation. Suggest removing this.

- This was suggested by the other reviewer. We changed this to a suggestion for future research.

Thanks for your very helpful reviews which greatly improved our manuscript. We hope that our revisions lead to a satisfactory paper which fits the standards of PLoS One.
